# The impact of long-term care insurance on the health status and healthcare expenditure of older adults in China

**DOI:** 10.3389/fpsyt.2024.1514603

**Published:** 2025-01-13

**Authors:** Ran An, Shujie Xiu, Xiaosheng Yang, Shishi Wang

**Affiliations:** ^1^ Department of Medical Insurance and Price, Dongguan First Hospital Affiliated to Guangdong Medical University, Dongguan, Guangdong, China; ^2^ School of Humanities and Manegement, Guangdong Medical University, Dongguan, Guangdong, China; ^3^ Medical Insurance Research Institute, Guangdong Medical University, Dongguan, Guangdong, China

**Keywords:** long-term care insurance, population aging, healthcare expenditures, health status, DID (difference-in-difference) model

## Abstract

**Objective:**

Long-term care needs have grown with population aging. This study explores the relationships among health status, healthcare expenditure, and long-term care insurance (LTCI) among the older adults.

**Methods:**

Using data from the China Health and Retirement Longitudinal Study (CHARLS 2011, 2013, 2015, 2018, and 2020) and based on the demand model for healthcare services, this study employed the difference-in-difference (DID) method to assess how the implementation of LTCI contributed to the health status and healthcare expenditures of the older adults. The propensity score matching DID method (PSM-DID) and other tests were used to conduct further robustness checks.

**Results:**

The findings demonstrate a significant positive impact of LTCI on improving the health status and reducing healthcare expenditures in the elderly population. The PSM-DID indicates that LTCI can effectively improve the health status of the elderly population and reduce their healthcare spending.

**Conclusions:**

Based on the findings, the development of relevant policy frameworks for LTCI in China is recommended. These include consider the differences among the various pilot cities and social strata to allow policy adjustments and improvements in a timely, establish a dynamic and diversified long-term care insurance financing mechanism, encourage collaboration between medical institutions and elder care facilities, establish effective contact between LTCI and medical institutions, and use incentive policies such as tax relief to provide financial support and subsidies.

## Highlights

- Long-term care insurance plays a pivotal role in enhancing the health status of the older adults in China.
- Long-term care insurance implementation leads to a reduction in the healthcare expenditure among the older adults.
- Long-term care insurance potentially alleviating financial burdens on individuals and society.
- Empirical evidence suggests that Long-term care insurance could be a cost-effective strategy for improving the well-being of the aged.
- Empirical evidence suggests that Long-term care insurance warranting further policy consideration and optimization.

## Introduction

The aging of the population has become an urgent problem worldwide. According to the National Bureau of Statistics, China has 297 million older adults aged 60 years or older, who accounted for 21.1% of the country’s total population by the end of 2023. By approximately 2035, China’s population aged 60 years and older will exceed 400 million, accounting for more than 30% of the total population, and the country will enter the stage of severe aging. Against the backdrop of rapid growth in life expectancy and a sharp decline in the fertility rate, the gap in care needs among the older adults is a serious challenge for China’s future (1). According to the fifth sample survey of the living conditions of the older adults in urban and rural areas, there are about 35 million disabled older adults in China, accounting for 11.6% of the elderly. The prevalence of the older adults is 4 times the average level of the total population, and the survival time with illness is more than 8 years. It is estimated that by 2035, the disabled older adults in China will reach 46 million. According to the region, the aging degree of the eastern and central regions is more than 50% of the aging population, about seven times the proportion of the older adults in western China. Increases in medical demand due to the aging population have led to a significant increase in medical costs. According to the China Health Statistics Yearbook (2023), China’s total medical and health expenditures were estimated to reach 9.05 trillion yuan in 2023, exceeding the total healthcare expenditures of the three years from 2011 to 2013, an increase of nearly 6 percentage points over 2022. Population aging is a major reason for this increase in total healthcare spending (2). Deterioration in health caused by increases in age results in greater use of health services among the older adults than at other life stages (3), and an increase in the proportion of older adults in the total population thus increases the overall use of health services and total healthcare spending.

Since the mid-20th century, European countries such as the Netherlands and Germany, as well as Asian nations such as Japan and South Korea, have successively established long-term care insurance (LTCI) systems to address the medical and healthcare care challenges brought about by an aging population. The long-term use of nursing care for older adults can effectively improve their health status (4), reduce the number of days spent hospitalized, and thus reduce healthcare expenditures (5, 6). LTCI tends to focus on providing care protection and financial compensation when the insured loses the ability to perform daily activities, becomes ill in old age, or passes away. In 2010, Chinese scholars, drawing on the experience of LTCI abroad, noted that establishing an LTCI system is an effective way to address the current increase in healthcare expenditures in China (7). In 2012, Qingdao took the lead in piloting LTCI in urban areas and became the first city in China to implement such a system; under the protection of LTCI, the level of health among the older adults in Qingdao significantly improved (8), and their healthcare spending fell (1, 9). People over the age of 60 who are disabled and continue for more than 6 months can apply for LTCI. In 2015, LTCI was included in China’s 13th Five-Year Plan, and a year later, China launched the first batch of LTCI pilot programs in 15 cities, including Shanghai, Guangzhou, Chengde, and Jingmen, which has made LTCI an important component of the country’s social security policy.

With the steady progress of the implementation of LTCI, its effects on the health status and healthcare spending of the older adults have garnered increasing attention and become a hot topic in the area of healthcare and social security. LTCI implementation effectively meets the care needs of older adults people, improves their activities of daily living (10), cognitive abilities (11, 12), mental health (13), and self-rated health status (8, 14), and significantly enhances their overall well-being (15, 16) and satisfaction (17). This has led to a notable increase in the overall health levels of the older adults (18, 19). Although there are various ongoing microlevel studies to improve the system after its introduction, policy impact analysis of the effect of LTCIs on the healthcare spending of older adults remains immature. Some studies have suggested that such spending decreased with the increase in the use of LTCI by older adults (20–22), while others pointed out that the role of LTCI in reducing healthcare expenditures is very limited (18) or may even increase healthcare spending (23, 24). There are also studies indicating that LTCI has both substitution and release effects on healthcare spending, because different healthcare approaches are tailored for older adults of different ages (25), in different cities (26), and of different types (27, 28).

In summary, examining the relationship between LTCI, health status of older adults, and healthcare expenditures is essential for enhancing senior health and ensuring appropriate care in later life. As a fundamental social security policy in China’s welfare-based elderly care system, LTCI is crucial for improving health outcomes for older adults and providing them with financially accessible care. This study contributes to the existing literature in three main aspects. First, previous research on LTCI has largely focused on individual cities, addressing caring needs and funding mechanisms, with limited attention to health outcomes and healthcare costs. There is a demand for empirical evidence on whether LTCI implementation across multiple pilot cities promotes better health status and reduces healthcare expenditures for older adults. This study addresses this gap. Second, this research develops theoretical models to examine how LTCI influences health status and healthcare expenditures among older adults. Using panel data from CHARLS spanning 2011 to 2020 and employing a difference-in-differences model, we assess the impact of LTCI on these outcomes. Robustness checks, including propensity score matching and additional tests, confirm our findings. The study also analyzes policy heterogeneity from individual and regional perspectives to further understand the LTCI-healthcare cost relationship. Third, the study’s findings support the refinement and broader implementation of LTCI in China to help address the societal challenges of aging. These insights also offer valuable guidance for other countries facing similar aging-related issues, particularly given China’s large population.

### Theoretical foundation

In the “Guiding Opinions on Expanding the Pilot Program for Long-term Care Insurance” issued by the National Healthcare Security Administration in 2020, LTCI was explicitly designated as the “sixth insurance” in social security (29). However, as a new insurance system, LTCI cannot avoid associations with other systems and policies, such as the integration of relevant systems, policy consolidation, and coordinated mechanisms. Simply comparing the outcome variables before and after policy implementation thus may not fully or accurately assess the factors influencing whether the LTCI policy improves or degrades the health status of the older adults or increases or decreases their healthcare spending. This paper therefore constructs a research framework based on the theoretical model of healthcare service demand and lays the foundation for subsequent empirical research.

Based on Mushkin’s (30) view of health as an investment and Becker’s (31) perspective of health as human capital, in 1972, Grossman (32) proposed the demand model for medical health services, which treats health as an investment good and explains changes in health status through individual health investment decisions. Health is seen as a consumer demand (or a commodity) and a raw material in which individuals can invest through healthcare consumption to yield healthy working hours (i.e., health as human capital); it also decreases with age or special events. The model suggests that individuals need to invest time, money, and other resources to maintain or improve their level of health. These investments are influenced by factors such as age, education level, and income level. According to this model, health is the core consumer demand, not just medical products or services—that is, the demand for medical products and services by older adults is based on pursuing and guaranteeing health. LTCI is a health security system for older adults and thus essentially provides a way for older adults to invest in health. As age increases, the physical functions of older adults gradually weaken, and their ability to invest in health autonomously also diminishes. In this context, LTCI can provide necessary medical care and support for older adults, help them improve their health status, and reduce healthcare expenditure. Within the framework of the Grossman model, LTCI can thus be seen as a means for older adults to increase their health investment. This paper therefore analyzes the impact of LTCI on the health status and healthcare spending of older adults based on the Grossman demand model for medical health services as the basic theoretical framework.

## Methods

### Data source and variable selection

The China Health and Retirement Longitudinal Study (CHARLS) is one of the most comprehensive and representative national longitudinal survey datasets on aging in China. It employs a multistage stratified sampling design and probability proportional sampling techniques to collect a high-quality set of microdata representing households and individuals aged 45 years and above. The data include information on personal details, family structure, health status, medical service use, and socioeconomic characteristics. It serves as the primary data source for this study.

Data from the five waves of the CHARLS—released in 2011, 2013, 2015, 2018, and 2020—were used for several reasons:

- The main beneficiaries of LTCI are middle-aged and older adults, and their interaction with the medical care system is most significant among older adults. CHARLS data focus on individuals aged 45 years and above, which aligns well with the needs of this study.
- This study investigated the impact of LTCI on the health status and healthcare spending of older adults. The CHARLS includes specific questions regarding residents’ health status and healthcare spending, thus providing the data necessary to support this research.

CHARLS data are publicly available, so this study does not require ethical approval.

The Ministry of Human Resources and Social Security’s “Guiding Opinions on Piloting Long-term Care Insurance” proposed establishing diversified funding channels for LTCI. Pilot cities have primarily employed three funding methods: fixed amount funding, proportional funding, and a combination of fixed and proportional funding. According to CHARLS data, 4 of the 15 pilot cities—Guangzhou in Guangdong Province, Jingmen in Hubei Province, Chengde in Hebei Province, and Shanghai—used proportional funding methods during the period 2016–2020. This study therefore designated these four pilot cities as the experimental group; after excluding the 11 other pilot cities designated in 2016, the remaining cities nationwide were used as the control group.

The data from 2011, 2013, and 2015 were combined to represent the period before the implementation of the LTCI policy, while the data from 2018 and 2020 were used for the period after policy implementation. Stata 18.0 software was employed for data computation and processing. After excluding cases with missing values for some basic variables and considering the robustness of the research results, the study winsorized the main variables to mitigate potential biases caused by extreme or outlier values, setting the winsorization proportion at 1%. The final effective sample consisted of 66,477 data points, with 2,701 in the experimental group and 63,776 in the control group.

### Dependent variables

Referencing current research, this study evaluated health status through measures of self-rated health status, life satisfaction, cognitive ability, and mental health. Healthcare expenditure levels were assessed by the number of years in the hospital, in-hospital out-of-pocket expenses, total cost of hospitalization, monthly outpatient number, out-of-pocket expenses and total outpatient expenses. Except for the specific indicators measuring healthcare expenditure levels, all other variables were categorized into five levels, with higher scores indicating better self-rated health or higher life satisfaction, ranging from 0 to 5. Inside, The cognitive ability consists of three items, five points for each item, and the scores of the three items are added together to form the final score, a total of 15 points. The higher the scores represent the stronger the cognitive ability. Mental health, in particular, was composed of 10 items, with higher scores indicating poor mental health.

### Independent variables

The core independent variable of this study was the policy effect of the LTCI (DID). When evaluating the impact of the LTCI on the health status and healthcare spending of the older adults, cities where the LTCI pilot policy had been implemented were designated the experimental group (City_ij_). If a city had implemented LTCI, a dummy variable was assigned the value of 1; otherwise, it was assigned 0. The years following the implementation of the LTCI pilot were considered post-event years (Post_it_) and assigned a value of 1; otherwise, they were given the value of 0.

### Covariates

The selection of covariates was based on Grossman’s health services demand theory model. According to previous research, gender, age, education status, registered permanent residence, and marital status were taken as individual demographic variables and per capita household consumption and annual household income as socioeconomic variables. Gender was coded as female (0) or male (1). Age was considered for individuals aged 60 years and above. Education status ranged from Below primary school (1) to High school and above (4). Registered permanent residence was coded as urban (0) and rural (1). Values for the variable “marital status” coded as married (0) to others (1). Indicators involving socioeconomic variables were recorded with specific numerical values.

### Baseline regression

The difference-in-differences (DID) model is an econometric method that has emerged for the quantitative evaluation of the effects of public policies or programs. This technique uses the actual changes in outcomes of a control group, which has not been subjected to the treatment, as a counterfactual to analyze the causal effects on the treated group’s outcomes had they not received the treatment (33). Tian et al. (12) applied this method to assess the impact of LTCI on the health status and health inequality among older adults; while Cao et al. (34) used it to investigate whether the implementation of LTCI could reduce disability among middle-aged and older adults individuals in China, while also examining the heterogeneity of its effects. The results indicated that the LTCI policy has had a positive impact on reducing disability among older adults in China.

Building on this foundation, the commonly used policy evaluation method, the DID approach, was adopted here to construct a regression model. This model was used to identify and test whether the implementation of the LTCI policy has influenced the health status and healthcare expenditures of the older adults. The DID model is specified as follows:


$$\begin{array}{c} Y_{ijt}=\alpha +\beta City_{ij}\times Post_{it}+\mu X_{ijt}+\tau_{t}+\omega_{i}+\epsilon_{ijt} \end{array}$$

where Y_ijt_ denotes a set of individual-level outcome variables, which include self-rated health status, life satisfaction, mental health, and cognitive ability as indicators of health status, and the number of annual hospital stays, out-of-pocket hospital costs, total hospital costs, monthly outpatient visits, outpatient out-of-pocket costs, and total outpatient costs as indicators of healthcare spending. City_ij_*Post_it_ represents the interaction term between the treatment group (the four selected pilot cities) and a dummy variable before and after the implementation of the LTCI, where the interaction term coefficient β is the core coefficient of this paper. This variable represents the difference in outcome variables between the pilot cities implementing the LTCI and other regions to explore the policy effects of the LTCI. Because the four selected pilot cities all started implementing the LTCI policy in 2016, the years 2011, 2013, and 2015 are set as before the treatment for the city samples (i.e., Post_it_=0), and 2018 and 2020 are set as after treatment (i.e., Post_it_=1). X_ijt_ represents a set of individual-level control variables, including demographic characteristics and socioeconomic characteristics; $\tau_{t}$ represents year fixed effects; $\omega_{i}$ represents individual fixed effects; and $\epsilon_{ijt}$ represents the error term; i represents the individual of the survey sample; j represents the region of the survey sample; t represents the time of the survey sample.

In summary, this study first conducts descriptive statistical analysis on the data from the experimental group to examine whether there are statistically significant differences in health status, healthcare expenditures, demographic characteristics, and socioeconomic features between the experimental and control groups. We then employ a DID approach to investigate the relationship between LTCI and the health status and healthcare expenditures of the older adults. To ensure the robustness of our findings, we perform several robustness checks, including parallel trends assumption, placebo tests, propensity score matching with DID, and balance diagnostics. Finally, to explore whether the health and economic effects of LTCI on the elderly population vary systematically, we conduct heterogeneity analyses based on individual and regional characteristics.

## Results

### Descriptive analysis

A descriptive statistical analysis was conducted on the selected indicators mentioned above. **Table 1** presents the descriptive analysis in health status and healthcare spending among the older adults of four cities—Guangzhou in Guangdong Province, Jingmen in Hubei Province, Chengde in Hebei Province, and Shanghai. The health status, healthcare spending, demographic characteristics, and socioeconomic characteristics on the experimental group were statistically significant. However, the extent of the impact of the LTCI policy requires further empirical analysis, as discussed in the following sections.

**Table 1:** Descriptive statistics of the variables (N=2701).

Major Variable	Definition	Fre.(%)	Mean	Std.
Dependent Variables				
Health Status				
**Self-rated health status**	1 very poor 2 poor 3 fair 4 good 5 very good	4.3 17.7 58.2 13.4 6.4	3.045	0.986
**Cognitive ability**	Cognitive score, from o to 15 (mean)	100.0	12.299	3.523
**Mental health**	depression score, from 0 to 10 (mean)	98.3	8.209	6.284
**Life satisfaction**	1 very poor 2 poor 3 fair 4 good 5 very good	94.9	3.225	0.781
Healthcare Expenditure				
**Number of years in the hospital**	Calculated according to the actual circumstance	92.4	0.279	1.029
**In-hospital out-of-pocket expenses**	Calculated according to the actual circumstance	87.6	938.882	7101.314
**Total cost of hospitalization**	Calculated according to the actual circumstance	87.6	1705.605	11620.142
**Monthly outpatient number**	Calculated according to the actual circumstance	92.0	0.499	1.530
**Out-of-pocket expenses**	Calculated according to the actual circumstance	86.6	169.870	1773.535
**Total outpatient expenses**	Calculated according to the actual circumstance	87.3	263.686	3911.058
Covariate				
**Gender**	0 female 1 male	51.8 48.2	0.475	0.499
**Age**	Calculated according to the actual circumstance	99.4	60.680	10.492
**Registered permanent residence**	0 urban 1 rural	35.4 64.6	0.765	0.424
**Educational status**	1 Below primary school 2 Primary school 3 Middle school 4 High school and above	32.2 28.5 21.4 17.6	2.019	1.056
**Marital status**	0 married 1 others	86.7 13.3	1.884	2.027
**Household per capita consumption**	Calculated according to the actual circumstance	95.1	14619.487	24819.285
**Household income**	Calculated according to the actual circumstance	93.9	37705.885	189481.571

Data source: China Health and Pension Tracking Survey, 2011, 2013, 2015, 2018, and 2020.

“Fre” represents Frequency Distribution; “Std” represents Standard Diviation.

### Benchmark regression results

**Table 2** shows that the coefficients for self-rated health status, cognitive abilities, mental health, and life satisfaction among the older adults in the four pilot cities are all significantly positive under the LTCI scheme. This further verifies that the implementation of LTCI has a significant positive impact on these variables among older adults in these four pilot cities, which in turn indicates that LTCI can effectively improve the health status of older adults, as shown by the results in the pilot cities.

**Table 2:** DID regression.

Dependent Variables	DID (Std. Err)	Dependent Variables	DID (Std. Err)
Self-rated health status	0.137*** (0.0186)	Number of years in the hospital	-0.115*** (0.0441)
Cognitive ability	0.450*** (0.0261)	In-hospital out-of-pocket expenses	-729.7*** (36.6700)
Mental health	-0.223*** (0.0061)	Total cost of hospitalization	-1,537*** (33.0600)
Life satisfaction	0.155*** (0.0259)	Monthly outpatient number	-0.961*** (0.2650)
		Out-of-pocket expenses	-125.4*** (15.6400)
		Total outpatient expenses	-108.7*** (13.9600)

*** p <0.01 indicate test levels of 1% respectively.

The coefficients for the healthcare spending indicators (e.g., number of annual hospitalizations, out-of-pocket hospitalization expenses, and total outpatient expenses) in these cities are all significantly negative. The implementation of LTCI thus appears to have a significant negative impact on the number of annual hospitalizations, out-of-pocket hospitalization expenses, monthly outpatient visits, out-of-pocket outpatient expenses, and total outpatient expenses among the older adults in the four pilot cities, which indicates that LTCI can effectively reduce healthcare spending for older adults.

### Robustness test

As demonstrated by the empirical analysis in the previous section, LTCI has a significant impact on the health status and healthcare spending of older adults in the four pilot cities of Guangzhou in Guangdong Province, Jingmen in Hubei Province, Chengde in Hebei Province, and Shanghai. LTCI implementation can thus effectively improve the health status of older adults and reduce their healthcare consumption. To validate the reliability of this conclusion, the four pilot cities—Guangzhou, Jingmen, Chengde, and Shanghai—were selected, and robustness tests were conducted on the empirical results.

### Parallel trend assumption

**Figure 1:**
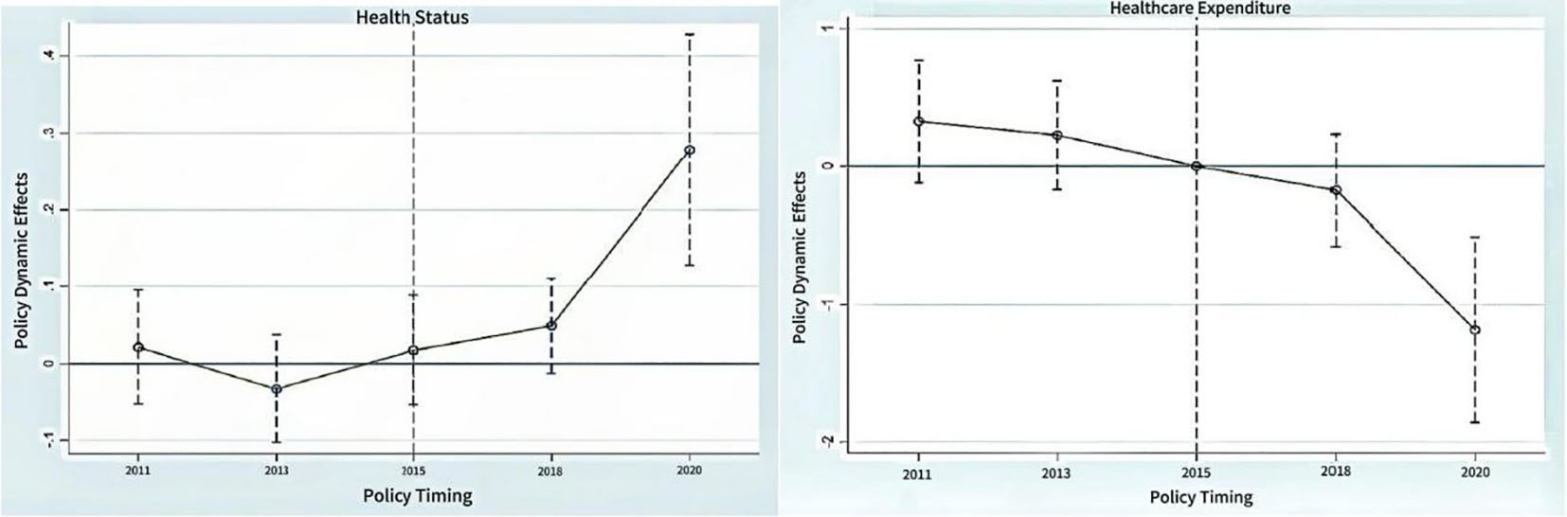
Dynamic effect of long-term care insurance policy.

The parallel trends test is an analytical method for assessing whether there is a correlation involving changes of the same magnitude between the data series of two variables. To better test the causal effect of the LTCI policy for improving older adults health status and reducing healthcare expenditures in the baseline regression analysis, it is necessary to conduct a parallel trends test on the DID model to further demonstrate the validity of the results. In **Figure 1**, the left figure presents the results of the parallel trend test for the DID model regarding health status, where 2011, 2013, and 2015 represent the three time points before policy implementation. From the figure, it is evident that the dynamic effect of the policy is not significant at these three time points, as indicated by the dashed lines crossing the zero point. This suggests that there were no notable changes in health status among the older adults during the period before policy implementation. Meanwhile, 2020 represents the time point after policy implementation, where the policy’s dynamic effect shows a significant positive impact, as indicated by the dashed lines no longer crossing the zero point. This suggests that the health status of the older adults has experienced a significant positive effect after the policy implementation.

Similarly, the right figure depicts the parallel trend changes in the level of healthcare spending before and after the implementation of the LTCI policy. The policy’s dynamic effects at the three time points of 2011, 2013, and 2015 are not significant, which indicates that there were no significant changes in the healthcare spending of the older adults before the policy was implemented. However, the dynamic effect of the policy at 2020 presents a significant negative impact, which reflects that the implementation of the LTCI had a significant negative effect on the healthcare spending of the older adults.

From the trend changes observed in **Figure 1**, the LTCI policy has had a very significant impact on both the health status and healthcare spending of the older adults in the four pilot cities.

### Placebo test

Another potential threat to the results of this study could be that changes in the health status and healthcare spending of the older adults might be attributable to cyclical policy changes. To eliminate potential confounding factors and enhance the credibility of the conclusions, a counterfactual testing method was employed. More specifically, multiple random sampling was conducted, fictitiously designating 2011 as the year of LTCI policy implementation and using it as the shock time for model regression. The reliability of the original estimation results is assessed by comparing the probability of the baseline regression estimation coefficients obtained from the fictitious experiment with the actual situation. If the fictitious estimation results are significant, this may suggest that the original estimation results are biased; conversely, if the fictitious estimation results are not significant, the original estimation results are relatively reliable. To further enhance the power of the placebo test, a distribution diagram of the estimation coefficients through 500 random samplings was drawn.

**Figure 2:**
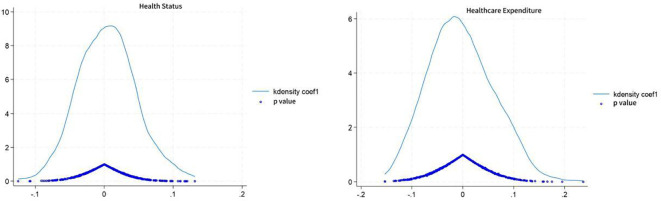
Virtual-time falsification test.

The density distribution of the regression coefficient is shown in **Figure 2**; the blue solid line represents the kernel density estimate, and the circles represent the corresponding *p* values. The estimates for health status and healthcare expenditures significantly deviated from the true estimates (0.96 and −0.502), which indicates that the analysis results influenced by random sampling are somewhat reliable. This further verifies that the changes in healthcare spending of the older adults were not triggered by cyclical policy changes. It can thus be inferred that the LTCI policy played a significant role in improving the health status and reducing the healthcare spending of the older adults in the four pilot cities. It also suggests that the previous empirical analysis results are robust and reliable.

### Propensity score matching DID

A propensity score matching differences-in-differences (PSM-DID) model can be used to assess the effectiveness of policies or interventions that combines PSM and the DID approach. During the baseline regression, because the experimental group and the control group are not randomly divided and there are differences in characteristics between the groups, selection bias may be a problem in the DID method. Such bias can cause a correlation between the independent variable and the residual term, leading to endogeneity issues. First, PSM was used to find a control group that is as similar as possible to the experimental group, and then DID was employed to obtain the average treatment effect of the policy. Finally, regression analysis was conducted on the matched samples. The specific results, as shown in **Table 3**, support the original theory presented in **Table 2**, thus confirming the robustness of our findings.

**Table 3:** PSM-DID regression.

Dependent Variables	PSM-DID (Std. Err)	Dependent Variables	PSM-DID (Std. Err)
Self-rated health status	0.149*** (0.0512)	Number of years in the hospital	-0.114*** (0.0436)
Cognitive ability	0.450*** (0.0261)	In-hospital out-of-pocket expenses	-729.7*** (36.6700)
Mental health	-1.477*** (0.4820)	Total cost of hospitalization	-1,818*** (75.7600)
Life satisfaction	0.322*** (0.1160)	Monthly outpatient number	-1.034*** (0.2660)
		Out-of-pocket expenses	-428.1*** (162.2000)
		Total outpatient expenses	-194.6*** (21.7300)

PSM-DID, Propensity score matching differences-in-differences; *** p <0.01 indicate test levels of 1% respectively.

### Balance diagnostics

**Figure 3:**
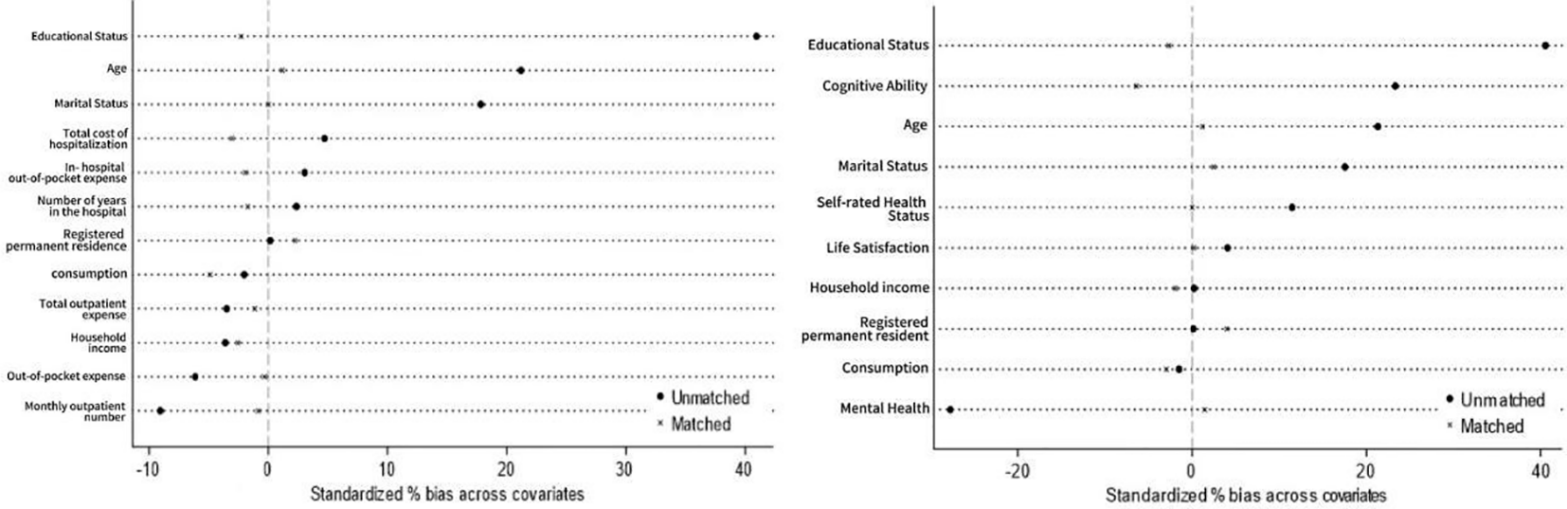
Balance test.

In conducting PSM, it is essential to make the distribution of covariates more uniform between the matched experimental and control groups to ensure accurate estimation. The test to assess the closeness of the mean covariates between the matched experimental and control groups is known as the balance test. **Figure 3** depicts the differences in the variables for health status and healthcare spending before and after matching, with the vertical line at 0 representing a standard bias of 0%. Both figures show a significant reduction in the standard differences of the covariates after matching, which are now concentrated at approximately 0; this indicates that the variables have achieved equilibrium and that the matching effect is good. This demonstrates that the matching variables and methods used in this paper are reasonable and effective.

### Heterogeneity analysis

The previous analysis revealed that LTCI has generally promoted the health status of the elderly and reduced their medical expenses. However, do these health and economic effects exhibit systematic differences based on individual or regional characteristics? To address this question, the present study conducted the following research.

First, focusing on the heterogeneity of individual characteristics, the sample was divided into two groups based on whether individuals are over 75 years old, younger elderly (coded as 0) and older elderly (coded as 1). A comparative analysis was then performed to gain deeper insights into the role of LTCI. As shown in **Table 4**, a DID empirical analysis was conducted based on age groups to evaluate the impact of LTCI on the health and medical expenses of the older adults. Overall, there are notable differences in how LTCI affects health levels and medical expenses across different age groups.

**Table 4:** Heterogeneity Test - Individual Characteristics.

	Younger Elderly Population	Older Elderly Population		Younger Elderly Population	Older Elderly Population
Dependent Variables	DID (Std. Err)	DID (Std. Err)	Dependent Variables	DID (Std. Err)	DID (Std. Err)
Self-rated health status	0.1460** (0.0577)	-0.1570* (0.0502)	Number of years in the hospital	0.0402 (0.0275)	-0.3360*** (0.0357)
Cognitive ability	0.4130*** (0.0500)	-0.4720*** (0.0512)	In-hospital out-of-pocket expenses	-169.3000 (282.4000)	-1,1260*** (92.6600)
Mental health	-0.2620*** (0.0197)	-0.1570* (0.0604)	Total cost of hospitalization	-1,0970* (368.4000)	-2,4640*** (148.9000)
Life satisfaction	0.1150 (0.0595)	0.0523*** (0.0014)	Monthly outpatient number	-0.2510 (0.1470)	-0.2430* (0.0894)
			Out-of-pocket expenses	-364.3000 (214.8000)	-93.0300*** (4.5960)
			Total outpatient expenses	-1,0970* (368.4000)	-174.2000*** (19.8100)

***p <0.01, **p <0.05, and *p <0.1 indicate test levels of 1%, 5%, and 10%, respectively.

In terms of health status, LTCI demonstrates a positive effect on the health levels of the younger elderly group, although it does not show a significant impact on their life satisfaction. For the older elderly group, while the effects are significant, SRH levels and cognitive abilities exhibit negative trends. Regarding medical expenses, the variables related to medical costs for the older elderly group show a significant negative correlation, indicating that the implementation of LTCI effectively reduces medical expenses in this demographic. In contrast, the negative impact on medical expenses for the younger elderly group is less pronounced.

By comparing the regression results from the two groups, it becomes evident that LTCI has a more substantial positive impact on the health status of the younger elderly, while it is more effective in reducing medical expenses for the older elderly. This discrepancy may arise because younger elderly individuals typically have better health and are more likely to benefit from preventive care; they generally possess better physical and cognitive functions, allowing them to adapt more readily to new care services and health interventions. However, as individuals age, their physical capabilities decline, and the risk of disability increases significantly, leading to a heightened demand for LTC. LTCI can integrate various caregiving resources to provide comprehensive care solutions, ensuring that older elderly individuals receive thorough and coordinated services, which can help avoid redundant medical services and unnecessary healthcare expenditures.

Secondly, regarding the heterogeneity of regional characteristics, the sample was divided into urban elderly (coded as 0) and rural elderly (coded as 1) based on their registered residence. A DID empirical analysis was conducted on these sub-samples, with results presented in **Table 5**. Overall, there are notable differences in the impact of LTCI on health levels and medical expenses across different regional groups.

**Table 5:** Heterogeneity Test - Regional Characteristics.

	Urban Elderly Population	Rural Elderly Population		Urban Elderly Population	Rural Elderly Population
Dependent Variables	DID (Std. Err)	DID (Std. Err)	Dependent Variables	DID (Std. Err)	DID (Std. Err)
Self-rated health status	0.0043 (0.1050)	0.1880* (0.0700)	Number of years in the hospital	-0.3330** (0.1100)	0.0862*** (0.0119)
Cognitive ability	0.3450 (0.2710)	0.8180*** (0.0232)	In-hospital out-of-pocket expenses	-1,5490** (322.1000)	-258.1000** (64.2700)
Mental health	-1.4080* (0.4970)	-1.8020*** (0.6220)	Total cost of hospitalization	-5,3010*** (297.0000)	-552.7000*** (87.7300)
Life satisfaction	0.1560 (0.0939)	0.2430*** (0.0250)	Monthly outpatient number	-0.1490 (0.1610)	-0.6720*** (0.2080)
			Out-of-pocket expenses	-192.4000 (99.2400)	-355.6000 (244.3000)
			Total outpatient expenses	305.0000 (287.2000)	-66.3000** (11.7800)

***p <0.01, **p <0.05, and *p <0.1 indicate test levels of 1%, 5%, and 10%, respectively.

In terms of health status, the rural elderly group showed significant improvements compared to their urban counterparts, indicating that LTCI effectively enhances the health conditions of the rural elderly. Regarding medical expenses, both urban and rural elderly groups demonstrated significant negative effects on the variables oophos1y and tothos1y. While the annual hospitalization frequency was also significantly correlated for both groups, the rural elderly exhibited a positive correlation. This may be attributed to the relatively poorer living conditions and healthcare facilities in rural areas compared to urban settings, which amplifies the beneficial impact of LTCI on their health.

## Discussion

In response to population aging, the General Office of the State Council of China issued the “Eleventh Five-Year Plan for Population Development and the 2020 Vision” in December 2006 to improve the social security system and promote coordinated socioeconomic development. For the first time, it proposed “exploring the establishment of socialized service systems such as volunteer services for older adults individuals, care savings, and long-term care insurance.” Over the following decade, policy documents were issued on the development of the elder care service industry and the integration of medical and nursing care. In 2016 and 2020, China successively launched two national pilot projects for LTCI in a total of 49 cities. This paper used CHARLS data and the DID method to analyze the impact of the LTCI policy, implemented in four pilot cities with a proportional financing method, on the health status and healthcare spending of older adults. Further robustness checks—including parallel trend tests, placebo tests, PSM-DID tests, and balance tests—were conducted to further evaluate the impact of the LTCI policy on these indicators.

First, the results of the descriptive statistical analysis suggest that the average health level in both the pilot cities and the remaining cities nationwide has improved, but the change in average health level has been more significant in the pilot cities. For example, the average self-rated health status of the older adults in the pilot cities increased from 2.96 to 3.96, while in the remaining cities, it increased from 3.02 to 3.03, an increase of merely 0.01. In terms of healthcare spending, all indicators showed a downward trend in the pilot cities, whereas the remaining cities nationwide exhibited only a slight downward trend for monthly outpatient visits.

Second, the implementation of LTCI can enhance the health level of the older adults and demonstrate a significant health effect, which is consistent with previous research. The empirical results indicate that all the health level indicators exhibited a positive and significant impact at the 1% level. The implementation of LTCI not only effectively increases the older adults positive assessment of their own health condition and improves their cognitive abilities but also provides financial support and daily care. Moreover, it significantly promotes their psychological health and effectively increases life satisfaction. By alleviating the economic and mental stress faced by older adults people when confronting life challenges, LTCI allows them to enjoy their later years with more peace of mind and comfort, thereby improving quality of life and well-being.

Finally, the implementation of LTCI can reduce the healthcare spending of the older adults through a substitute effect, which aligns with most findings from prior research. The empirical results show that the implementation of LTCI could reduce the annual number of hospitalizations by 0.12 times, the out-of-pocket hospitalization expenses by 729.70 RMB, the total hospitalization costs by 1537 RMB, the monthly number of outpatient visits by 0.96 times, the outpatient out-of-pocket expenses by 125.40 RMB, and the total outpatient costs by 108.70 RMB for older adults individuals in the four pilot cities. LTCI can thus effectively reduce the use of medical resources and improve the efficiency of nursing and medical resource use, thereby decreasing unnecessary healthcare behaviors and healthcare spending.

In summary, LTCI implementation has a positive effect on improving the health status of the older adults and reducing healthcare spending. However, the specific effects of its implementation need to be evaluated and adjusted based on conditions present in different regions and countries. At the same time, it is also necessary to consider the potential challenges and issues facing the LTCI system to make timely adjustments and improvements.

## Conclusion

Based on our research findings, it is crucial to sustain the development of the LTCI service policy system in our country. First, efforts should be made to accelerate the establishment of a nationwide unified institutional foundation, enhancing the level of centralization to improve operational efficiency and address sustainability issues. A dynamic and diversified LTCI financing mechanism should be established, further exploring a multi-source financing model that includes contributions from individuals, enterprises, social organizations, and the government. Regarding the base and rates for financing, a dynamic adjustment mechanism should be implemented. Second, the government should consider the differences among various pilot cities and social strata to make timely adjustments and improvements to policies, addressing the challenges and evolving needs brought about by population aging. It is essential to establish effective linkages between LTCI and healthcare institutions, promoting resource sharing and complementary advantages. This will provide older adults with more convenient, efficient, and comprehensive medical, nursing, and elderly care services. Promoting and encouraging collaboration between healthcare institutions and elderly care facilities will facilitate the development of an integrated model of medical and elderly care, thereby strengthening the long-term care service system. Finally, the government should adopt incentive policies such as tax reductions to strengthen financial subsidies. Diversified fiscal subsidies should be used to augment the national social security fund, and the investment scope of the fund should be moderately expanded to enhance the pension insurance reserves. Adhering to the principles of mutual aid and shared responsibility, the government should encourage participation from both enterprises and individuals in LTCI, thereby reducing the overall insurance costs.

### Limitations

When studying the influencing factors of health status and healthcare expenditures in the older adults, socioeconomic factors, mental health, lifestyle, and environmental conditions are also included besides physiological and pathological factors that directly affect health. For example, some studies have shown that medical and health service factors, genetic and other biological factors, lifestyle and environmental factors jointly affect the health level of the older adults, among which the contribution rate of lifestyle and environmental factors is nearly 80%. In addition, psychosocial factors such as social support system, education level, marital status, and career history were also considered as important influencing factors. Before conducting specific research on the influencing factors of older adults health status and healthcare expenditures, this paper uses statistical models, such as the Tobit model, to examine the effects of different factors on the health status and healthcare expenditures of older adults, and identify significant influencing factors such as chronic diseases, income, residence, and social security. Finally, in the process of selecting long-term care recipients, we cannot circumvent the question of assessing whether the severity of neurological disease would introduce selection bias, which is a current difficulty in this study.

## Data Availability

Publicly available datasets were analyzed in this study. This data can be found here: https://charls.pku.edu.cn/.
